# Genome-Wide Detection of Allele Specific Copy Number Variation Associated with Insulin Resistance in African Americans from the HyperGEN Study

**DOI:** 10.1371/journal.pone.0024052

**Published:** 2011-08-25

**Authors:** Marguerite R. Irvin, Nathan E. Wineinger, Treva K. Rice, Nicholas M. Pajewski, Edmond K. Kabagambe, Charles C. Gu, Jim Pankow, Kari E. North, Jemma B. Wilk, Barry I. Freedman, Nora Franceschini, Uli Broeckel, Hemant K. Tiwari, Donna K. Arnett

**Affiliations:** 1 Department of Epidemiology, University of Alabama at Birmingham, Birmingham, Alabama, United States of America; 2 Department of Biostatistics, University of Alabama at Birmingham, Birmingham, Alabama, United States of America; 3 Division of Biostatistics, Washington University, St. Louis, Missouri, United States of America; 4 Department of Biostatistical Sciences, Wake Forest School of Medicine, Winston Salem, North Carolina, United States of America; 5 Department of Epidemiology, University of Minnesota, St. Paul, Minnesota, United States of America; 6 Department of Epidemiology, University of North Carolina, Chapel Hill, North Carolina, United States of America; 7 Carolina Center for Genome Sciences, University of North Carolina, Chapel Hill, North Carolina, United States of America; 8 Department of Neurology, Boston University, Boston, Massachusetts, United States of America; 9 Department of Internal Medicine/Nephrology, Wake Forest School of Medicine, Winston Salem, North Carolina, United States of America; 10 Department of Medicine, Medical College of Wisconsin, Milwaukee, Wisconsin, United States of America; Universita Magna-Graecia di Catanzaro, Italy

## Abstract

African Americans have been understudied in genome wide association studies of diabetes and related traits. In the current study, we examined the joint association of single nucleotide polymorphisms (SNPs) and copy number variants (CNVs) with fasting insulin and an index of insulin resistance (HOMA-IR) in the HyperGEN study, a family based study with proband ascertainment for hypertension. This analysis is restricted to 1,040 African Americans without diabetes. We generated allele specific CNV genotypes at 872,243 autosomal loci using *Birdsuite*, a freely available multi-stage program. Joint tests of association for SNPs and CNVs were performed using linear mixed models adjusting for covariates and familial relationships. Our results highlight SNPs associated with fasting insulin and HOMA-IR (rs6576507 and rs8026527, 3.7*10^−7^≤*P*≤1.1*10^−5^) near ATPase, class V, type 10A (*ATP10A*), and the L Type voltage dependent calcium channel (*CACNA1D,* rs1401492, *P*≤5.2*10^−6^). *ATP10A* belongs to a family of aminophospholipid-transporting ATPases and has been associated with type 2 diabetes in mice. *CACNA1D* has been linked to pancreatic beta cell generation in mice. The two most significant copy variable markers (rs10277702 and rs361367; *P*<2.0*10^−4^) were in the beta variable region of the T-cell receptor gene (*TCRVB*). Human and mouse TCR has been shown to mimic insulin and its receptor and could contribute to insulin resistance. Our findings differ from genome wide association studies of fasting insulin and other diabetes related traits in European populations, highlighting the continued need to investigate unique genetic influences for understudied populations such as African Americans.

## Introduction

Long term insulin resistance is the major precursor to type 2 diabetes (T2D), a condition which currently affects more than 10% of Americans older than 20 years of age [1], [2]. Insulin resistance is a complex disorder influenced by lifestyle and genetic factors where the natural hormone, insulin, becomes less effective at lowering high blood sugar. When a compensatory increase in insulin secretion does not occur, blood glucose concentrations increase and set the stage for progression to T2D [3]. While there are various tests used to detect insulin resistance, fasting insulin levels and the Homeostasis Model Assessment Insulin Resistance Index (HOMA-IR) are widely used in population based studies [4]. Based on these measures, the risk of developing insulin resistance and subsequent T2D varies between different ethnic populations and is at least two times greater among African Americans compared to Caucasians [3].

African Americans have been severely under-represented in genome wide association studies (GWAS) of T2D and/or related traits [5]. There is a need for such studies as replicated variants derived from Caucasian samples have not consistently generalized to T2D related risk in African Americans [6]–[8]. This could be due to different disease pathology with unique genetic risk variants amongst African Americans or reflect differences in allele frequencies or patterns of linkage disequilibrium [6], [9]–[11].

Current generation single nucleotide polymorphism (SNP) genotyping arrays have been designed to assay copy number variants (CNVs), a type of structural variant which may explain additional genetic variability associated with complex human traits [12], [13]. A recent report from the Wellcome Trust Case Control Consortium questioned the added value of analyzing common copy number variation (that can be genotyped on existing platforms) in genome wide studies of SNPs. Specifically, they identified several CNV loci that are associated with common disease (including T2D) and showed those CNVs were generally well tagged by nearby SNPs [14]. However, those conclusions were based on data from populations of European ancestry. Thus, it is unclear if CNVs (assayed on existing platforms) are well tagged by SNPs in African American populations given higher degrees of recombination and shorter spans of linkage disequilibrium in comparison to European populations [9].

Methods using the software package *Birdsuite* have been developed to concurrently analyze SNP and CNV polymorphisms assayed on the Affymetrix 6.0 platform [15]. Additionally, research has effectively shown targeting quantitative traits in normoglycemic populations in GWAS searches can help identify genetic determinants of overt disease [16]. Therefore, in this study we performed joint tests of association between SNPs and CNVs at the genome-wide level with fasting insulin and HOMA-IR in 1,040 African American participants without diabetes in the Hypertension Genetic Epidemiology Network (HyperGEN) study.

## Methods

### Ethics Statement

The study was approved by the University of Alabama at Birmingham's Internal Review Board for Human Use, the Washington University Human Research Production Office, the University of North Carolina's Office of Human Research Ethics and the Medical College of Wisconsin's Office of Human Research Protection Program. All subjects provided written informed consent. The study conforms with the principles outlined in the Declaration of Helsinki, and all procedures followed were in accordance with institutional guidelines.

### Study Population

Study participants were enrolled in the HyperGEN (Hypertensive Genetic Epidemiology Network) study. HyperGEN is part of the Family Blood Pressure Program funded by the National Heart Lung and Blood Institute and was designed to study the genetics of hypertension and related conditions. Participants were recruited from multiply-affected hypertensive sibships ascertained through population-based cohorts or from the community-at-large. The study was later extended to include siblings and offspring of the original sibpair. Probands were identified by the onset of hypertension before age 60 and the presence of at least one additional hypertensive sibling who was willing to participate. Participants with type 1 diabetes or advanced renal disease (defined as serum creatinine level >2 mg/dL) were excluded from the original study since these two conditions can cause secondary hypertension and the goal of HyperGEN was to identify novel essential hypertension loci. Recruitment, cross-sectional clinical measurement, and DNA isolation were completed in 2003. Two of four centers (AL, NC) recruited 1,264 African Americans, while three centers (NC, MN, and UT) recruited Caucasians [17]. Persons reporting treatment with insulin or oral hypoglycemic agents were excluded from the current study (N = 212).

### Laboratory Measures

Insulin measured after at least 8 hours of fasting was recorded for all participants in HyperGEN. Samples were collected in a resting state for insulin concentration using an automated immunoassay instrument (Beckman Coulter, Fullerton, CA, USA) [18]. HOMA-IR was calculated as (fasting insulin x (fasting glucose x 0.056))/22.3, with glucose in the units of mg/dL, and insulin in the units of µIU/L. The measurement of fasting glucose in HyperGEN has been described [19].

### Genotyping

DNA extraction and purification from HyperGEN has been described in Williams *et al*. (2000) [17]. Genotyping was performed using the Affymetrix Genome-Wide Human SNP 6.0 Array following the Affymetrix defined protocol. Genotypes were called using *Birdsuite*, an open-source set of tools developed at the Broad Institute [15]. *Birdsuite* is multi-stage analytical framework that determines allele specific copy number calls at each locus. The *Larry Bird* application in *Birdsuite* generates integrated SNP/CNV genotype calls ranging from 0–4 copies of each allele.

### Quality Control

A total of 909,622 markers were assayed. The median marker call rate was 99.97%. The overall Mendelian inheritance error rate was 0.04%. We removed SNPs due to excessive missingness (>1%; N = 282), low minor allele frequency (<1%; N = 21,875), or evidence for deviation from Hardy-Weinberg equilibrium in founders (*P*<0.0001; N = 15,222). All marker locations are based on the hg18 reference genome assembly.

While the error rates for SNP genotypes are typically small (<1%), the corresponding rates for array-based detection of CNV are considerably larger and dependent on the CNV size and frequency [15], [20]. Thus, we only considered CNVs with frequency >1% and, based on the *Birdsuite* developer's recommendations, confidence values less than 0.1 in this report [15].

### Statistical Methods

Genetic admixture can both confound population association studies [21] or produce spurious results [22]–[25]. As a proxy for the degree of global European ancestry, in our African American population we generated principal components (PCs) from all 872,243 SNPs using *Eigenstrat* [26]. Based on the examination of the scree plot [27] (Figure S1) the first four PCs were retained and used as covariates for all association analyses.

Joint tests of association for allelic and copy number variation with fasting insulin levels and HOMA-IR were conducted on all 872,243 autosomal SNPs passing quality control thresholds. These joint models were analogous to that proposed by Korn *et al.,* with the exception of including random effects for each family [28]. Because it was not computationally feasible to fit a linear model including random effects for all 872,243 loci, we performed a two-stage procedure using the genome-wide rapid association using mixed model and regression (GRAMMAR) approach introduced by Aulchenko *et al* [29]. In stage 1, residuals measuring the log-transform (to achieve normality of residuals) of measures of insulin resistance were generated via a mixed model after controlling for relatedness and fixed effect covariates. In terms of the present study:
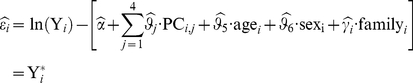
(1)where, 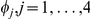 are the fixed effects from the first four principal components obtained from *Eigenstrat* to control for admixture, 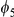 and 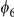 are the fixed effects for age and sex, and 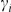 is the family random effect of the 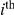
subject [26]. Residuals in equation 1 were obtained from the PROC MIXED procedure in SAS® software, Version 9.2 (SAS, Cary NC). The resulting residuals (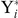) for the 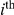
subject were then regressed against his or her allelic and copy number state at each locus in a linear model:
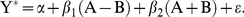
(2)In equation 2, A is the number of A alleles at the locus, B is the number of B alleles at the locus, 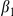 corresponds to an additive allelic effect, while 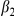 represents an additive copy number effect. The joint test involves assessing the null hypothesis,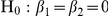. When either SNP allele or copy number state is invariant, the model defaults to a single test of either allelic or copy number effect: 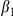 = 0 or 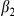 = 0, respectively. Analyses of the residuals in equation 2 was performed via *lm* function using R version 2.8.0 (R development core team, 2008).

In stage 2, the markers corresponding to the top 1,000 most significant findings from stage 1 were then re-analyzed using the full mixed model (for the log-transform of Y) [30]. The full mixed model used for analysis can be written as:
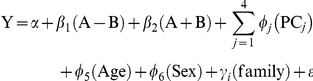
(3)The parameters in equation 3 correspond to those described above. Models in the second stage were obtained using the *lmer* function in R.

The UCSC Genome Browser was used to locate genes within 200 kilobases (kb) up or downstream of loci that were significantly associated with our phenotypes [31]. The Gene Sorter program was then used to identify expression patterns, homology and other information on the identified genes [32].

## Results

The mean age of the population was 43.5±13 years, the majority of participants were female (66%) and recruited in Alabama (79%). The mean fasting glucose in our study population was 95±16 mg/dL (5.2±0.9 mmol/L). The average fasting insulin measure was 10.2±8 µIU/mL (70.8±55 pmol/L) and 5% of the study population had fasting insulin surpassing the upper level of the normal range (3–25 µIU/mL) [33], [34]. The average fasting HOMA-IR was 2.5±2 (no units).

The first stage of the GRAMMAR procedure conservatively tests the hypotheses of association [29]. For each outcome the first stage results obtained for all 872,243 loci were used as a screening tool to identify possible associations. From these results, the 1,000 markers with the smallest *P*-values were tested in the second stage. These markers were fit into the full model described in Equation {3}. In **Table 1** we report the most significant markers derived from the full model for each outcome. For both fasting insulin and HOMA-IR the most significant findings were not copy number variable and the model defaulted to a model of SNP effects. Detailed results for all markers analyzed in the second stage analysis are presented in **Table S1**. Fasting insulin and HOMA-IR were positively correlated with r = 0.96 and we found several markers with evidence of association with each trait (**Table S1**). Two markers highlighted in Table 1 (rs1401492 and rs6576507) were among the top results for both traits. Overall, none of the observed markers reached the typically utilized threshold for genome-wide significance (*P*<5*10^-8^). Nonetheless, our analyses do highlight some biologically interesting regions with suggestive evidence of association.

**Table 1:** Top loci associated with fasting insulin [ln(µIU/mL)] and HOMA-IR in African Americans without diabetes from HyperGEN.

Marker†	Chr	A_1_ ‡/A_2_	MAF	Gene*	% effect (95% CI)	*P*-Value
**Insulin**						
rs6576507	15	A/G	0.13	ATP10A	14 (8,20)	4*10^−7^
rs8026527	15	A/G	0.13	ATP10A	14 (8,20)	6*10^−7^
rs12655917	5	C/T	0.01	AP3B1	−40 (−51, −27)	6*10^−7^
rs4819143	21	C/T	0.06	*PCBP3*	−17 (−23, −10)	1*10^−6^
rs17431357	12	C/T	0.01	TRIAP1	−34 (−44, −22)	1*10^−6^
rs2407103	8	C/T	0.09	KCNU1	15 (9,23)	2*10^−6^
rs1401492	3	C/T	0.07	*CACNA1D*	−15 (−21, −9)	3*10^−6^
rs591044	22	A/G	0.37	*SEZ6L*	9 (5,13)	6*10^−6^
rs604459	22	C/T	0.38	*SEZ6L*	8 (5,12)	8*10^−6^
**HOMA-IR**						
rs7043482	9	G/T	0.19	-	12 (7, 18)	3*10^−6^
rs17589516	6	C/T	0.02	ZFAND3	45 (24, 69)	4*10^−6^
rs9792548	9	C/T	0.15	GAS1	−12 (−16, −7)	4*10^−6^
rs1401492	3	C/T	0.07	*CACNA1D*	−16 (−22, −9)	5*10^−6^
rs16962638	13	C/T	0.11	-	15 (8, 22)	6*10^−6^
rs6576507	15	A/G	0.13	ATP10A	13 (7, 20)	7*10^−6^
rs2407314	8	C/G	0.47	*CSMD1*	−9 (−12, −5)	8*10^−6^

† For more detailed information about these markers see Table S1.

‡ A_1_ is the modeled and minor allele.

*nearest gene within a 200 kb window on either side of the marker (if gene is italicized the marker lies in the gene).

Among the markers we analyzed, 35% were identified as copy number variable in this dataset. For fasting insulin we identified 13 markers where the CNV effect showed a trend toward significance (*P_CNV_*≤9*10^−4^). However, most of these associations were influenced by CNVs at low frequency in the cohort (<1%). Results for HOMA-IR were similar. As this is the first GWAS of insulin resistance in African Americans to include analysis of copy number variation that we are aware of, we highlight frequent (>1%) copy variable markers with *P_CNV_*≤9*10^−4^ in **Table 2**. The copy number variable region on Chromosome 7q34 associated with both outcomes is interesting for follow-up. Two nearby markers (rs10277702 and rs361367) are informed for copy number variation by 21 surrounding probes. Both markers correspond to a deletion present in 23 participants and both CNVs are positively associated with fasting insulin and HOMA-IR.

**Table 2:** Top copy variable loci associated with fasting insulin [ln(µIU/mL)] and HOMA-IR in African Americans without diabetes from HyperGEN.

Marker†	Chr	A_1_ ‡/A_2_	Gene*	Effect	Freq‡	% Effect	*P*-Value
**Insulin**							
rs10277702	7	C/T	*TCRVB*	SNP	0.49	−1 (−4,3)	0.57
				CNV	0.02	−46 (−61, −27)	1*10^−4^
				Joint			4*10^−4^
rs361367	7	C/T	*TCRVB*	SNP	0.42	0 (−4,3)	0.81
				CNV	0.02	−45 (−60, −25)	2*10^−4^
				Joint			8*10^−4^
rs12552047	9	C/G	-	SNP	0.01	−25 (−36, −12)	3*10^−4^
				CNV	0.01	−47 (−64, −23)	9*10^−4^
				Joint			1*10^−4^
**HOMA-IR**							
rs10277702	7	C/T	*TCRVB*	SNP	0.49	−2 (−5, 2)	0.39
				CNV	0.02	−52 (−66, −33)	3*10^−5^
				Joint			8*10^−5^
rs361367	7	C/T	*TCRVB*	SNP	0.42	−1(−5, 3)	0.57
				CNV	0.02	−52 (−66, −31)	6*10^−5^
				Joint			2*10^−4^
rs13003829	2	A/G	ARHGEF4	SNP	0.21	1 (−4, 5)	0.77
				CNV	0.02	−47 (−63, −26)	3*10^−4^
				Joint			1*10^−3^
rs12509348	4	A/G	DCK	SNP	0.17	−3 (−8, 2)	0.25
				CNV	0.02	74 (25, 240)	9*10^−4^
				Joint			1*10^−3^

† For more detailed information about these markers see Table S1.

‡ A_1_ is the modeled/minor allele for which frequency (freq) is reported.

*nearest gene within a 200 kb window on either side of the marker (if gene is italicized the marker lies in the gene).

## Discussion

Using the Affymetrix Genome-Wide Human SNP Array 6.0 we analyzed the joint effect of SNPs and CNVs with measures of insulin resistance in 1,040 African American participants from the HyperGEN study that had no personal history of diabetes. The majority of GWAS findings to date highlight variants associated with fasting glucose and diabetes status rather than fasting insulin and HOMA-IR levels [16], [35], [36]. We found no evidence of association of fasting glucose or diabetes related loci from recent GWAS in our study of insulin resistance in African Americans from HyperGEN. Variants associated with fasting insulin (and HOMA-IR) with significance at the genome wide level in European populations include the glucokinase regulatory protein gene (*GCKR*) and insulin-like growth factor 1 (*IGF1*). In our study, markers in these genes were not associated with fasting insulin or HOMA-IR (*P*>0.05) nor did any of our top hits from Table 1 replicate in a large cohort (N>40,000) of persons of European ancestry from the MAGIC consortium (*P*>0.2) [16]. While some loci, e.g., transcription factor 7-like 2 (*TCF7L2*), are associated with diabetes and related traits across racial populations, the majority of studies, including ours, have not replicated risk loci across these two racial groups [6]–[8], [37].

In agreement with published GWAS [16], [35], [36] the magnitude of effect for markers of interest (**Table 1**) is generally small [16], [35]. However, we found some biologically plausible associations that approached significance in our study. Among the top hits shared by both traits are loci (rs6576507, rs8026527) 180 kb downstream of ATPase, class V, type 10A (*ATP10A*). *ATP10A* belongs to the subfamily of aminophospholipid-transporting ATPases. One study reported an unusual pattern of maternal inheritance of the chromosomal region containing the gene (also referred to as *ATP10c*) was associated with obesity, T2D, and nonalcoholic fatty liver disease in mice [38]. The marker rs1401492 is highlighted in Table 1 for both traits and lies in intron 3 of the L Type voltage dependent calcium channel *CACNA1D* (chr3∶53,504,071–53,821,532). *CACNA1D* has been shown to be induced by a high fat diet [39] and required for pancreatic beta cell generation in mice [40]. Other interesting markers highlighted in Table 1 include rs12655917 and rs17431357 for fasting insulin just upstream (<6kb) of the beta 1 subunit of the adaptor-related protein complex 3 (*AP3B1*) and the of p53-inducible cell-survival factor (*TRIAP1*), respectively. *AP3B1* is involved in the subcellular trafficking of vesicular cargo proteins [41]. It is lowly expressed in pancreatic islets (where insulin secreting beta cells reside) and, could play a role in insulin secretion [32]. *TRIAP1* prevents induction of apoptosis and is expressed in the pancreas and islets. Pancreatic beta-cell death by apoptosis contributes significantly to both autoimmune type 1 diabetes and type 2 diabetes [42]. Top results for HOMA-IR include markers near growth arrest specific gene 1 (*GAS1*) and CUB and Sushi multiple domains 1 (*CSMD1*). *GAS1* is a cell cycle inhibiting protein that has been linked to cancer and *CSMD1* has been associated with hypertension and peripheral artery disease in GWAS of persons of Asian descent [43]–[45].

The effect size for copy variable markers presented in **Table 2** (on the order of 30–40%) is similar to the effect size for SNPs of the same frequency (MAF 1–2%) from **Table 1**. The markers rs10277702 and rs361367, most significantly associated with both outcomes, lie in the well characterized T-cell receptor beta variable region (*TCRVB*, chr7∶141,695,793–142,058,637) [46]. The copy number variable regions of T-cell receptors (TCR) have been sequenced for a variety of autoimmune diseases. One study reported that TCR from humans with diabetes and non-obese mice with diabetes mimic insulin and the insulin receptor. These data could explain how insulin, and its receptor are targets of autoimmunity in diabetes [47]. This data are interesting for follow-up given the accumulating evidence of immune system involvement in the pathophysiology of type 2 diabetes [48] and provide a potential link to the pathology of insulin resistance in African Americans.

This study population was ascertained on hypertension status and antihypertensive treatment has been associated with changes in glucose homeostasis [49]. In our study population, 15%, 25% and 33% were treated with an ACE inhibitor, calcium channel blocker and diuretic, respectively, and these variables were associated with our measures of insulin resistance (*P*<0.1). Treatment with a beta blocker or alpha blocker was not associated with either outcome. Additionally, insulin resistance is strongly associated with obesity through common pathways such as inflammation [50]. Thus, we considered models adjusted for body mass index and the antihypertensive treatments described aiming to uncover novel findings and/or stronger effects for discovered variants. The results are presented in **Table S2** and largely show adjusting for these additional factors uncovered association with many of the same markers as reported in this manuscript (and **Table S1**) with decreased significance. For example, rs8026527 and rs6576507, the top hits for fasting insulin (*P*<6*10^−7^), were also positively associated with fasting insulin for the fully adjusted model with *P*<2*10^−5^.

We implemented the GRAMMAR method for quantitative traits specific for family data described by Aulchenko *et al.* [29] where genetic association is first tested using a screening model. The screening model included an analysis of genotypes with a set of residuals derived from a model of the phenotype adjusted for non-genetic factors. We then re-analyzed the most significant loci derived from the screening model using the full model adjusted for genetic and non-genetic factors. Aulchenko *et al.* [29] reported that the analysis of the residuals alone for a quantitative trait is conservative compared to the full model and supported the re-analysis of the full model for markers that reach a pre-defined significance threshold. Our results support use of this two-stage procedure. For example Figure S2 is the Quantile-Quantile (QQ) plot of observed versus expected *P*-values from the screening model for fasting insulin and portrays how the residual models do not reach the expected significance under the null hypothesis for a wide range of markers. We chose, *a priori*, to re-analyze 1,000 loci with the full model and could have missed interesting markers with this approach. However, we felt this approach was conducive to isolate potentially significant loci while preserving computational efficiency. Finally, this study considered additive models of inheritance which have been proposed as the most powerful approach for the purposes of gene discovery in GWAS [51]. Future larger studies of replicated genetic effects may further interrogate alternate modes of inheritance of risk loci useful for predictive modeling and/or functional experiments [52].

Genomic studies hold the promise for early identification of risk for insulin resistance and subsequent progression to type 2 diabetes. To our knowledge, this is the first GWAS of insulin resistance in an African American population that combines SNP and CNV data. The markers we identified have not been noted in GWAS of European populations [16], [35]. However, the most significant SNP and CNV loci highlighted in our study are near genes (*ATP10A*, *TCRVB*) that have been identified as playing a role in T2D *in vivo*. We stress the need to replicate these findings in additional African American cohorts with subsequent functional experiments to fully characterize replicated effects. In conclusion, this study highlights several interesting genomic regions associated with measures of insulin resistance in the understudied African American population. Future studies are needed to continue to dissect the genetic architecture of diabetes and related traits in African Americans.

## Supporting Information

Figure S1 **Eigenvalues associated with principal components generated in Eigenstrat from genetic data on 1,040 African Americans from HyperGEN.**
(TIF)

Figure S2 **Fasting Insulin Quantile-Quantile (QQ) Plot generated from the stage 1 analysis screening model.**
(TIF)

Table S1 **Expanded results for top 1000 loci associated with fasting insulin [ln(µIU/mL)] and HOMA-IR in African Americans without diabetes from HyperGEN.** Models are adjusted for age, sex, genetic admixture and family relationship.
(XLS)

Table S2 **Expanded results for top 1000 loci associated with fasting insulin [ln(µIU/mL)] and HOMA-IR in African Americans without diabetes from HyperGEN.** Models are extended from Table S1 to be additionally adjusted for antihypertensive treatment (ACE inhibitor, calcium channel blocker, diuretic) and BMI.
(XLS)
