# Supplementary figures and images for: Genome-Wide Detection of Allele Specific Copy Number Variation Associated with Insulin Resistance in African Americans from the HyperGEN Study
Source: PLoS One. 2011 Aug 25;6(8):e24052. doi: 10.1371/journal.pone.0024052 (PMC3162025; doi:10.1371/journal.pone.0024052)

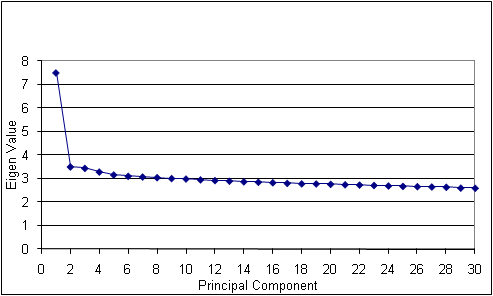

Supplement: Figure S1 — Eigenvalues associated with principal components generated in Eigenstrat from genetic data on 1,040 African Americans from HyperGEN. (TIF) [file pone.0024052.s001.tif]
